# Parathyroid Carcinoma Mimicking a Parathyroid Adenoma on [^18^F]F-Choline-PET With Occult Metastases

**DOI:** 10.1097/RLU.0000000000006555

**Published:** 2026-06-17

**Authors:** Carola M. Bregenzer, Roman Trepp, Florian Dammann, Reto M. Kaderli, Aziz Chouchane, Robert Seifert

**Affiliations:** * Department of Nuclear Medicine, Inselspital, Bern University Hospital, University of Bern, Bern, Switzerland; † Department of Diabetes, Endocrinology, Nutritional Medicine and Metabolism, Inselspital, Bern University Hospital and University of Bern, Bern, Switzerland; ‡ Department of Diagnostic, Interventional and Pediatric Radiology, Inselspital, Bern University Hospital, University of Bern, Bern, Switzerland; § Visceral Surgery and Medicine, Inselspital, Bern University Hospital, University of Bern; ∥ Institute of Tissue Medicine and Pathology, University of Bern, Bern, Switzerland

**Keywords:** [^18^F]F-choline-PET/CT, [^18^F]FDG-PET/CT, parathyroid adenoma, parathyroid carcinoma

## Abstract

An 80-year-old man with hypercalcemia and hyperparathyroidism was suspected of having a parathyroid adenoma, which was localized on [^18^F]F-choline-PET/CT. Still, after surgical removal of the suspected adenoma with an intraoperative parathyroid hormone decrease of >50%, severe hypercalcemia recurred quickly. A repeated [^18^F]F-choline-PET/CT raised suspicion of a single contralateral parathyroid adenoma or multiglandular disease. Given the inconclusive findings, a histological reassessment of the suspected parathyroid adenoma showed the presence of a singular vessel invasion, which alerted the diagnosis to parathyroid carcinoma. An additionally performed [^18^F]FDG-PET/CT revealed a liver lesion and MRI confirmed multiple liver metastases, which could not be seen on [^18^F]F-choline-PET/CT.

**FIGURE 1:**
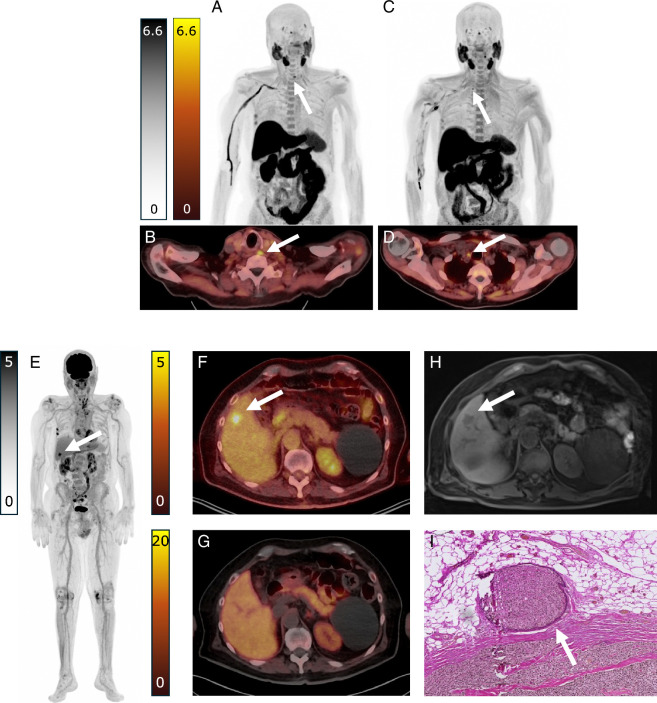
An 80-year-old man with weakness, weight loss of 15 kg in 2 months, hypercalcemia, and hyperparathyroidism underwent [^18^F]F-choline-PET/CT because of suspected parathyroid adenoma. Color bars with standard uptake values (SUV) are presented on the left side of the figures. The maximum intensity projection (MIP) (**A**) as well as the fused axial slice (**B**) of the [^18^F]F-choline-PET/CT show a focal uptake dorsal of the left thyroid lobe (marked with arrow) consistent with an imaging-derived diagnosis of a parathyroid adenoma. After surgical removal, pathology assessment confirmed the presumptive diagnosis of parathyroid adenoma. However, the quick recurrence of hypercalcemia, hyperparathyroidism, and severe symptoms prompted another [^18^F]F-choline-PET/CT to rule out missed multiple adenomas. The repeated PET showed a new focal uptake in the right paratracheal area (MIP image **C**, fused axial slice **D, marked with arrow**), which was suspected to represent another previously marked parathyroid adenoma or a reactive lymph node. No residual uptake could be seen on the left side. Due to persistently high parathyroid hormone values, an [^18^F]FDG-PET/CT was performed, which showed a focal uptake in the liver (MIP **E marked with arrow**) in segment V (fused axial slice **F , marked with arrow**) without [^18^F]F-choline avidity (**G**). Contrast-enhanced liver MRI confirmed the liver lesion (**H, marked with arrow**) and detected multiple additional liver metastases, not previously detected on low-dose, nonenhanced CT nor on PET/CT ([^18^F]FDG and [^18^F]F-choline). A concurrent reevaluation of the initial pathology sample revealed a singular vessel invasion (**I, marked with arrow**), altering the initial diagnosis to parathyroid carcinoma. Postoperative hypercalcemia after resection of a suspected singular parathyroid tumor with elevated parathormone levels can be caused by missed multiglandular disease.^1^ Parathyroid carcinoma is a rare cause for hyperparathyroidism (0.8%–4.6%)^2^ with an incidence per year of around 5.73/10.000.000,^3^ which can result in postoperative persistent hyperparathyroidism due to metastatic disease. Even though parathyroid adenomas can be detected on [^18^F]F-choline-PET/CT with a sensitivity >90%,^4^ imaging of parathyroid carcinoma seems challenging. The usefulness of [^18^F]F-choline-PET/CT for detecting the primary parathyroid carcinoma was reported.^5^ Even metastasized parathyroid carcinomas, with metastases in the skull base,^6^ in the brain,^7^ in the bone, and lungs^8^ could be detected by [^18^F]F-choline-PET/CT. For local recurrence, a case was reported without any differences between [^18^F]FDG-PET/CT and [^18^F]F-choline-PET/CT.^9^ Other authors suggest a dual tracer protocol with [^18^F]FDG and [^18^F]F-choline.^8^ Our case demonstrates 2 relevant findings: first, a solitary lesion on [^18^F]F-choline suspected to represent a parathyroid adenoma can, in rare cases, instead be parathyroid carcinoma. Second, metastatic lesions of parathyroid carcinoma may be missed when relying only on [^18^F]F-choline for imaging. Therefore, dual-tracer protocols should be considered in cases where results are inconclusive.
